# Retraction notice to “Cognition enhancing effects of *Clausena lansium* (Lour.) peel extract attenuate chronic restraint stress-induced memory deficit in rats” [Heliyon 7 (2021) e07003]

**DOI:** 10.1016/j.heliyon.2025.e42493

**Published:** 2025-02-05

**Authors:** W. Phachonpai, T. Tongun

**Affiliations:** a Division of Physiology, School of Medical Sciences, University of Phayao, Phayao, 56000, Thailand; b Department of Physiology, Faculty of Medicine, Khon Kaen University, Khon Kaen, 40002, Thailand

This article has been retracted: please see Elsevier Policy on Article Withdrawal (https://www.elsevier.com/locate/withdrawalpolicy).

This article has been retracted at the request of the Editor-in-Chief.

The article is a duplicate of a paper that has already been published in Journal of Applied Pharmaceutical Science, Volume: 10, Issue: 7, July, 2020, DOI: 10.7324/JAPS.2020.10703. Redundant publications overweigh the relative importance of published findings and distort the academic record of the authors. One of the conditions of submission of a paper for publication is therefore that authors declare explicitly that the paper has not been previously published and is not under consideration for publication elsewhere. As such this article represents a misuse of the scientific publishing system.

The scientific community takes a very strong view on this matter and apologies are offered to readers of the journal that this was not detected during the submission process.

